# Development of Functional Models for a SOD

**DOI:** 10.1155/MBD.2002.9

**Published:** 2002

**Authors:** Ali Arslantas

**Affiliations:** 1 Department of Chemistry Kafkas University Kars TR 36100 Turkey; 2 K.T.B.K. 13 Nolu A Tipi Gida Kontrol Müf. K. Girne Northern Cyprus Turkey

## Abstract

Superoxide dismutase (SOD) is the scavenger of superoxide anion (O2^−^) and functions as a protector of living bodies. Study of a model compound of SOD is important when searching for the relationship between functions and structures of enzymes. Furthermore, SOD model compounds have potential for therapeutic usefulness. Although many SOD: model compounds have been reported, their structures are quite different from those of the native enzyme. Cu,Zn-SOD has been proposed for clinical uses. Unfortunately, many problems such as half-lifetime and antigenicity have not been overcome even though several copper(II) complexes are known to show SOD activity. Active oxygen species such as superoxide (O2^−^) from various components of the cellular electron transport chains, and provided during the respiratory burst of phagocytic cells, have been implicated both in the aging process and in degenerative diseases, including arthritis and cancer. Therefore, the biological system posseses the protective mechanisms against active species.


### Development of Functional Models For a SOD

Ali Arslantas

*Department of Chemistry, Kafkas University, Kars, Turkey ,TR 36100**

### ABSTRACT

Superoxide dismutase (SOD) is the scavenger of superoxide anion ($O_2^-$) and functions as a protector of living bodies. Study of a model compound of SOD is important when searching for the relationship between functions and structures of enzymes. Furthermore, SOD model compounds have potential for therapeutic usefulness. Although many SOD model compounds have been reported, their structures are quite different from those of the native enzyme. Cu,Zn-SOD has been proposed for clinical uses. Unfortunately, many problems such as half-lifetime and antigenicity have not been overcome even though several copper(II) complexes are known to show SOD activity. Active oxygen species such as superoxide ($O_2^-$), being formed by leakage of electrons to oxygen ($O_2$) from various components of the cellular electron transport chains, and provided during the respiratory burst of phagocytic cells, have been implicated both in the aging process and in degenerative diseases, including arthritis and cancer. Therefore, the biological system posseses the protective mechanisms against active species.

**Abbreviations:** Cu,Zn-SOD; copper, zinc-containing superoxide dismutase; SOD, superoxide dismutase; Mn-SOD; manganese-containing superoxide dismutase; Fe-SOD, İron-containing superoxide dismutase.

### INTRODUCTION

The reactive superoxide radical anion, $O_2^-$, is a product of the oxygen metabolic cycle /1/. The radical anion is a highly reactive toxic species in many biological systems. Superoxide dismutase (SOD) catalyses $O_2^-$ dismutation very efficiently and it serves as an important means of defense against oxygen toxicity. It has been discovered /2/ that the superoxide dismutase enzymes catalyze disproportionation of the toxic superoxide ions into molecular oxygen and $H_2O_2$ (Eq. 1).

$$2O_2^- + 2H + SOD \rightarrow O_2 + H_2O_2 \quad (1)$$

* Present address: K.T.B.K. 13 Nolu A Tipi Gida Kontrol
Müf. K. Girne, Northern Cyprus, Turkey.

The *in vivo* ubiquity of the SODs makes them very efficient in normal conditions. However, in the case of an oxygen burst (during reperfusion following ischemia, for instance) the natural defenses of the organism are insufficient, leading to lipid peroxidation, membrane damage, and cell death. These effects are not directly due to the superoxide anion, but rather to the more potent oxidant, the hydroxyl radicals, which is generated *in situ*. In order to make up for the SOD deficiency, the first idea was to introduce supplementary SODs into the organism. However, SODs have molecular weights too high to cross cell membranes /3/ and can only provide extracellular protection /4/. In order to circumvent this difficulty, low-molecular mass synthetic compounds that mimic SODs have been investigated /5/. As copper has been proven to be the active metal center in the best studied SOD (Cu,Zn-SOD), many cuprous complexes have been synthesized and tested for SOD-like activity /3, 5, 6/ and most them appeared to be very efficient. The problem is that they lose their activity *in vivo* /3, 7/. Proteins appear to have better affinities for copper than the studied ligands, so Cu is inactivated once it is embedded in the proteins. The other two classes of SODs, which contain iron or manganese, have received less attention, and their structures have only recently been described /3, 8/. However, some Fe /7, 9, 10/ and Mn /7, 9, 11/ SOD mimics have been reported, and some of them show a marked SOD activity and seem to keep it in living cells /10, 11/.

From all these results on native SODs or low molecular weight SOD mimics, it seems that the presence of coordination sites belonging to nitrogen heteroaromatic rings such as imidazoles or pyridines is important to have high SOD activity that is not affected by biological chelators /3, 10/. The problem to solve when searching for SOD-like complexes is to find a balance between a sufficient stability necessary to survive *in vivo* conditions and a certain flexibility that allows the change of metal coordination occuring during the catalytic process. Apart from the use of various metalloporphyrins /3/, only a few mono- /12/ and bitopic (13) macrocyclic Cu (II) complexes have been investigated as potential SOD-like derivatives, but as acyclic Cu (II) complexes, they do not resist biological chelators. Recently, two Mn (II) macrocyclic complexes have been shown to exhibit catalytic SOD activity maintained *in vivo* conditions (13). Many research groups have been pursuing the possibility of developing such "synzymes" (synthetic enzymes) as an approach to managing various types of diseases. Tremendous progress has been made in this area in recent years, both in defining a role for such a synthetic enzyme as a human pharmacological agent by utilizing a number of animal models for disease, and in progressing toward development of actual drug candidates. The following review briefly introduces the chemistry of the SOD enzymes, surveys recent advances in the synthesis of low molecular weight SOD mimics, and attempts to introduce some of the issues involved with the testing for SOD activity and the chemical design constraints one must satisfy in order to synthesize a highly active enzyme mimic which can function as a human pharmaceutical agent. In particular, emphasis will be made in this review on considerations of development of functional models for a SOD for the metal complexes reported to possess SOD synzyme activity and recent developments in the chemistry of SOD which may have implications for these areas.

### MECHANISTIC ASPECTS

The major function of superoxide dismutase (SOD) is to catalyze the dismutation of superoxide anions ($O_2^-$) and to control intracellular and extracellular concentrations of $O_2^-$ /14/. $O_2^-$ is the mediator of many diseases. It is involved in radiation injury, DNA damage, lipid peroxidation and vascular diseases /15, 16/. Although SOD has been considered for application as a pharmaceutical for many years /17/, some problems have been encountered in clinical trials, because SOD has some disadvantages such as high cost, instability, cell impermeability and immunogenicity. Therefore, the stable, non-toxic, low molecular weight metal complexes (model compounds) which catalyze the dismutation of $O_2^-$ have attracted much attention /18-23/. It is of great importance to study the structures, thermodynamic and kinetic properties of SOD model compounds and their mechanism of catalytic dismutation of $O_2^-$ when searching for relationships of structures, properties and functions and medical uses of metal complexes /24, 25/. Until now several Cu (II) complexes for mimicking $Cu_2Zn_2SOD$ have been described, such as Cu (II) complexes of polypeptides /26, 27/, polydentate Schiff bases /16, 28/, mixed ligand /12, 29/, imidazolate-bridged heterobinuclear Cu-Zn complexes /30, 31/, and complexes of macrocyclic ligands /31, 5/. Some of them were shown to mimic the structures of $Cu_2Zn_2SOD$ to different extents, and others can mimic their functions.

Reactive oxygen species such as superoxide anion, hydroxyl radical, hydrogen peroxide, and single oxygen have been postulated as playing an important role in a wide variety of pathological processes /32/. Cu,Zn-superoxide dismutase (SOD), which controls reactive oxygen species via disproportionation of $O_2^-$ radicals into $O_2$ and $H_2O_2$, has been proposed for clinical uses /33, 34/ . Unfortunately, intravenously injected SOD disappears from the circulation with a half-life of the order of some minutes /35/. Most SOD research has been directed at prolonging the half-life and, of course, maintaining full enzymatic activity /36/. Even if substantial advances have been made in the development of SOD derivatives that enhance lifetimes /37, 38/, the limiting factor in the use of such compounds, antigenicity, has not been overcome /39/. A variety of low molecular weight SOD mimics have been prepared, both as antioxidants and pharmaceutical agents. Manganese /17/, iron /40/, or copper ions /5/ either free or complexed, are known as efficient catalysts of the dismutation process. A variety of copper complexes of 1, 10-phenanthroline, amino acids, peptides, salicylates, macrocycles, and Schiff base derivatives have been verified as catalysts for superoxide dismutation /41, 12/. Even if SOD mimics, based on complexed copper, can be stoichiometric rather than catalytic scavengers of oxygen radicals, copper, once freed from the complexity agent, might catalyze hydroxyl radical formation. Furthermore, some potent effects of copper (II) compounds with antioxidant activity have been registered /42, 43/. Recently, kinetic and thermodynamic considerations have shown that Cu, Zn-superoxide dismutase is unique in its ability to catalyze $O_2^-$ dismutation *in vivo* in contrast to copper compounds which have this feature *in vitro*, due to different reactivity towards dioxygen (low for SOD and high for copper complexes) /44/. If, by means of this contribution, it is suggested that copper compounds may efficiently replace SOD only in those pathological processes in which the local concentration of $O_2^-$ can be rather high, it is not clear which parameters are involved in the differing dismutation ability shown by different copper complexes *in vitro*. Although some hypotheses have been put forward /45, 46/, there is some doubt that the proposed activity-structure relationships are correct. In fact, not considering that copper forms labile complexes, the scavenger activity is attributed to the same species obtained in the solid state as well as dissolved in the reaction medium. In a medium where competitive ligands such as buffer, xanthine, and $OH^-$ are present; this type and concentration of copper compounds can change, invalidating any structure-activity correlation. Recently, Costanzo *et al.* have found that some copper complexes have a productive effect against photohemolysis sensitized by 2-(3-benzoylphenyl) propionic acid (ketoprofen) /15/, a drug which undergoes photodegradation involving a superoxide radical as a reactive intermediate /47/. Site-directed mutagenesis is an important tool in studying and understanding the factors determining enzymatic mechanisms and the role of the residues involved in the catalytic reaction /48, 49/. Substitution of some of the potentially critical residues in the active site cavity of $Cu_2Zn_2$ superoxide dismutase (SOD hereafter) has recently led to a better understanding of their role in the catalytic mechanism /50, 51/.

SODs are, in the majority of cases, cytoplasmic enzymes, predominantly found in eukaryotes, which protect cells against the toxicity of superoxide, a by product of aerobic metabolism. SOD is a very efficient catalyst for the dismutation of superoxide to molecular oxygen and hydrogen peroxide /2, 52/. Moreover SOD is a dimer of identical subunits, each of them containing one copper and one zinc ion /53/, the copper ion being essential for the catalytic reaction. The efficient catalytic properties of SOD depend on the redox potential of the $Cu^{+2}/Cu^{+}$ pair, which is intermediate between the potentials of the pairs $O_2$ /$O_2^-$ and $O_2/O_2^{-2}$. During the catalytic reaction, the copper ion is cyclically reduced and oxidized with the consequent production of $O_2$ and $H_2O_2$ respectively /54/. Both reactions have rate constants of about $2 \times 10^9$ $S^{-1}$ $M^{-1}$ /55/; such high rates are thought to be due to increased substrate attraction towards the active channel due to positive electrostatic field at its entrance /55/.

### CuZn-SUPEROXIDE DISMUTASE

CuZn-SOD is a metalloprotein which catalyzes the scavenging of superoxide anion $O_2^-$ (Eq. 2) /56/.

$$2O_2^- + 2H^+ + \text{Cu-SOD} \rightarrow H_2O + O_2 + \text{Cu-SOD} \quad (2)$$

The catalytic site is composed of a copper (II) ion ligated by four histidines and water molecule in a distorted five-coordinated geometry and a Zn (II) ion ligaTed by three histidines and an aspartate in a distorted tetrahedral environment /57/. Only the copper (II) ion undergoes oxidation-reduction cycling during the dismutation of $O_2^-$ /58/. The zinc ions are not involved in the redox cycle, but maintain the configuration of the active site and facilitate the oxidation step /59, 60/. In recent years particular attention has been paid to synthetic analogs of Cu, Zn-SOD /61, 62/. In this perspective, the coordination structures and redox potentials of each Cu (II) ion in the synthesized copper (II) complexes which can be considered to possess SOD-mimic activity were found to play a significant role /57/. Copper-zinc-superoxide dismutase (Cu-Zn-SOD) is believed to protect cells from the toxic effect of superoxide ion by catalyzing the dismutation reaction of $O_2^-$ /2, 11/. X-ray crystal structure analysis shows that the active site of Cu, Zn-SOD from bovine erythrocytes comprises two identical subunits, each of which contains a histidine (imidazolate)-bridged Cu (II)-Zn (II) active center. In Cu-Zn-SOD /63/, the subunits contain fully active imidazolate-bridged bimetallic centers in which Cu (II) has replaced Zn (II).

Numerous imidazolate-bridged binuclear copper (II) complexes have been prepared and characterized as models for Cu, Zn-SOD active site. Two imidazolate-bridged mononuclear complexes proved to be insufficient as models because their imidazolate bridge in aqueous solution is stable only in a very narrow pH range. In order to obtain imidazolate-bridged binuclear copper (II) complexes that are stable over a larger pH range, several imidazolate-bridged dicopper (II) complexes with macrocyclic ligands have been reported /64, 65/ and been considered to be better Cu, Zn-SOD (or Cu, Cu-SOD) mimics due to their enhanced stability by the presence of the binucleating macrocycles or macrobicycles. Two of these cycles seem to be better ligands for the imidazolate bridge to accommodate in: one is the 24-membered alicyclic hexaazamacrocycle /64/ and the other is the 24-membered octaazamacrocycle with three xyls /31/.

One of these metalloenzymes is Cu, Zn-SOD which is a dimeric protein (MW=31200) with two identical subunits, each containing one $Cu^{+2}$ and one $Zn^{+2}$ ion. The direct utilization of this natural enzyme as a pharmaceutical agent is limited because of low membrane permeability as a consequence of its high molecular weight /66/. Therefore, considerable efforts were made in order to obtain nontoxic, low molecular weight biomimetic molecules, which are able to catalyze the dismutation of superoxide anion and therefore to provide a suitable alternative to superoxide dismutase in clinical application /17/. A variety of low molecular weight complexes of transition metals, especially those of copper, were prepared and studied as SOD mimics /64, 12/. Examples of such complexes include derivatives of the antiinflammatory drugs salicylates, amino acids, peptides, and amines /12/.

Valproic acid (2-propylpentonoic acid) in the form of its sodium salt, $(CH_3CH_2\ CH_2)2CHCOO^-Na^+$, has a wide spectrum of activity as an anticonvulsant drug /67/. The observation that copper (II) complexes of anticonvulsant and antiinflammatory drugs are more active agents than the activity of such drugs may be due to the *in vivo* formation of metallic complexes /68/. Physical studies of copper (II) valproate /69/ have shown that it contains binuclear units with bridging carboxylate ligands similar to other copper (II) carboxylates /70/. Several binuclear copper (II) carboxylates of antiinflammatory drugs such as salicylates /12/, indomethacin /71/, and lonazoloc /72/ were studied as SOD mimics, but not of anticonvulsant drugs such as valproate. In addition, it was reported that the presence of coordination sites belonging to nitrogen heteroatomic rings such as imidazoles or pryridines is important for SOD activity /5/. And since complexes with bipyridines and phenathrolines are DNA intercalators, showing an ability to inhibit nucleic acid synthesis *in vivo* /73/, these ligands are used in this study to form ternary copper (II) valproate complexes.

### Mn-SUPEROXIDE DISMUTASE

The Mn- and Fe-containing superoxide dismutases (SODs) are a family of enzymes which is widely dispersed in microorganisms /74/. Two types of superoxide dismutases have been distinguished. The first type includes those SODs for which the apoenzymes are active with only one of the metals: manganese or iron, and replacement of that metal by the other results in loss of activity. For example, the *E. Coli* MnSOD is active with manganese, but not with iron /75/, and FeSOD from *P. ovalis* is active with iron only /76/. The second type includes those SODs which are active with both metals. Such enzymes have been termed "cambialistic SODs" /77/, an example being the SOD isolated by Matsumoto *et al.* /78/, which was found to be active either with manganese or iron. Iron and manganese superoxide dismutases from bacterial sources have been shown by several crystallographic studies to be structural homologs. Their active sites of the iron and manganese SODs share the same metal ligands, with similar ligand coordination geometries /74/. The metal cofactor specificity of some enzymes is unclear in light of the structural similarities, but it is reasonable to suppose, as it was suggested previously by others /74/, that the immediate environment of the metals in the enzymes must differ.

The isolation of MnSOD from the $Ba_1$ bacterium was reported /74/. This is a moderately halophilic halotolerant microorganism which can withstand large variations in external salt concentrations. The enzyme contains an inactive iron, which differs in its environment from the iron in *E. coli* FeSOD. The presence of manganese and iron in SOD from $Ba_1$ raises the question of their role in the enzyme's activity. The fact that $H_2O_2$, known to inactivate FeSODs /79, 80/, did not affect the activity of SOD from $Ba_1$ does not contribute to clarify their activity in the enzyme.

### SUPEROXIDE DISMUTASE ACTIVITY

The superoxide dismutase-mimetic activity of the binary $Cu_2(valp)_4$ complex and their ternary complexes with diimines was studied using the alkaline $Me_2SO$-NBT method /12, 80/. A unit superoxide dismutase activity is the concentration of complex or enzyme which causes 50% inhibition of alkaline $Me_2SO$-mediated reduction of NBT; this concentration is expressed as IC50 for comparative purposes. The data for SOD mimetic activity of the complexes under investigation are shown in Table 1 /80/. In addition, to ascertain the effectiveness of the present complexes as functional SOD mimics, a comparison was made /80/ of the IC50 of several known Cu (II) complexes (Table 1), which were previously demonstrated as SOD mimics /12/, by the NBT method under the same conditions. The data suggest that the activities of the present complexes are higher than those of other copper (II) complexes. The mechanism believed to be operating in both Cu, Zn-SOD and Cu (II) complexes involves the initial binding of superoxide to be axial Cu (II) site, with subsequent redox cycling of the Cu (II) ion (Eq. 3 and 4) /64/.

$$Cu^{+2} + O_2^{-} \rightarrow Cu^{+} + O_2 \qquad (3)$$

$$Cu^{+} + O_2^{-} + 2H^{+} \rightarrow Cu^{+2} + H_2O_2 \qquad (4)$$

Some factors were suggested which may discriminate among the dismutation features of the copper (II) complexes *in vitro*, and these may include: First a fast exchange of molecules axially linked to the center and a limited steric hindrance to the approach of the superoxide anion are considered essential requirements for the successful binding of the $O_2^-$ radical /80/. Second the flexibility of the copper (II) arrangement, which facilitates the interaction of the $O_2^-$ radical, followed by the rapid electron transfer reaction which results in reduction to copper (II)-$O_2^-$ species /32/. Third the favorable response of $\pi$-electrons of the coordinated ligands in stabilizing the Cu(II)-$O_2^-$ interaction /7/.

The control of the free radical flux derived from oxygen is jeopardized in many circumstances in which superoxide (SO) anion production is excessive. This overproduction of SO can overwhelm the body's ability to catalytically dismute superoxide and reduce or eliminate the radical burden. This deleterious oxygen-derived free radical has been demonstrated to be a mediator of reperfusion diseases, such as those following

**Table 1**

Superoxide dismutase-Mimetic Activity /80/

| Compound | IC50[a]/ μm |
|---|---|
| $Cu_2(valp)_4$, (1) | 10.4 |
| $(Cu(valp)_2(2,2^{\prime}\text{-bpy}))\ H_2O$, (2) | 4.2 |
| $Cu(valp)_2(phen)$, (3) | 4.5 |
| $Cu(valp)_2(dmph)$, (4) | 6.3 |
| $(Cu(valp)_2(\mu\text{-}4,4^{\prime}\text{-bpy})_n$, (5) | 18.3 |
| $(Cu_2(valp)_4(\mu\text{-}4,4^{\prime}\text{-bpy}))_n$ (6) | 5.0 |
| $Cu(salicylate)_2$ | 44 |
| $Cu(aspirinate)_2(pyridine)_2$ | 13 |
| Cu(glycylglycinate)(2,2' -bpy), $3H_2O$ | 25 |
| Cu(glycylglycinate)(phen), 3H2O | 32 |
| $Cu(cimetidine)^2(ClO_4)2$ | 4.0 |
| Cu,Zn-SO | 0.72 |

[a]I50 is defined as the concentration of complex or enzyme which produces 50% inhibition of NBT reduction.

acute myocardial infarct or stroke, and shown to be associated with development and continuation of inflammatory processes, involved in diseases such as arthritis, and to play a major role in the initiation of neurological disorders such as Parkinson's disease /81/. Given the high reactivity of the superoxide radical, however, it was hypothesized that Cu, Zn-SOD might also associate with the membrane surfaces of mitochondria and peroxisomes, both of which generate substantial amounts of this radical. To test this hypothesis the subcellular localization of Cu, Zn-SOD was examined in rat brain and liver as well as in cultured human fibroblasts with the use of antibodies specific for Cu, Zn-SOD, Mn-SOD. This test provided direct evidence that Cu, Zn-SOD is associated with both mitochondria and peroxisomes in the brain, liver, and fibroblasts /82/. Higher organisms produce superoxide anion as an occasional byproduct during the one-electron reduction of dioxygen; this occurs in respiration and photosynthesis. Also, in animals, macrophases generate superoxide as part of the immune response. Organisms must therefore have ways to regulate superoxide concentrations since excess amounts can inactivate enzymes containing iron-sulfur clusters and can lead to the formation of highly oxidizing species damaging to other cellular constituents /83/. Recently, the three-dimensional structure of Cu, Zn-SOD from *photobacterium leiognathi* has been reported and interesting differences with respect to the eukaryotic SODs have been described concerning the dimer interface region and the assembly of the electrostatic loop forming the active site /84/. This enzyme catalyses a very rapid two-step dismutation of superoxide to dioxygen and hydrogen peroxide through an alternate reduction and oxidation of the active-site copper ion /85/.

### CONCLUSION

Our vast knowledge of superoxide dismutase (SOD) chemistry and biology is moving the field towards new and exciting directions. There are detailed issues relating to the mechanism and its biological functions that are being addressed. It is important to note that these enzymes are involved in the biosynthesis of metabolites, which form the largest pool of compounds from which numerous pharmaceutical substances have been discovered. We can expect new and exciting developments in this area in the near future. Several model studies have advanced our understanding of the interacting SOD in biological systems.

### REFERENCES

1. I. Fridovich, *Science* **201**, 825 (1978); D. Salvemini and R. Botting, *Drug News Perspect.* **6**, 274 (1993).
2. J. M. Mccord and I. Fridovich, *J. Biol. Chem.* **244**, 6049 (1969).
3. A. Gartner and U. Weser, in *Topics in Current Chemistry*, F. Vogtle and E. Weber, Eds., Springer, Berlin, **132**, 1 (1986).
4. W. Huber, M. G. P. Saifer and L. D. Williams, in *Biological and Clinical Aspects of Superoxide Dismutase*, W. H. Bannister and J. V. Bannister, Eds., Elsevier, New York, **11B**, 395 (1980).
5. E. Bienvenue, S. Chona, M. -A. Lobo-Reciu, C. Marzin, P. Pacheco, P. Seta and G. Tarrago, *J. Inorg. Biochem.* **57**, 157 (1995)
6. S. Goldstein and G. Czapski, *J. Am. Chem. Soc.* **105**, 7276 (1983).
7. A. Bury, A. E. Underhill, D. R. Kemp, N. J. O'Shea, J. P. Smith and P. S. Gomm, *Inorg. Chim. Acta* **138**, 85 (1987).
8. A. Bury, A. E. Underhill, M.B. Fleet, P.J. Keymer, A. Stevens and P. S. Gomm, *Inorg. Chim. Acta* **158**, 181 (1989).
9. C. J. Bijloo, H. Van der Goot, A. Bast and H. Timmerman, *J. Inorg. Biochem.* **40**, 237 (1990).
10. R. G. Bhirud and T. S. Srivastava, *J. Inorg. Biochem.* **40**, 331 (1990).
11. S. Goldstein, G. Czapski and D. Meyerstein, *J. Am. Chem. Soc.* **112**, 6489 (1990).
12. R. G. Bhirud and T. S. Srivastava, *Inorg. Chim. Acta* **179**, 125 (1991).
13. J. Labuda, J. Kratsmar-Smogrovic, M. Vanickova and L. Gazova, *Chem. Papers* **45**, 605 (1991).
14. A. E. Underhill, S. A. Bougourd, M. L. Flugge, S. E. Gale and P. S. Gomm, *J. Inorg. Biochem.* **52**, 139 (1993).
15. L. L. Costanzo, G. DeGuidi, S. Giuffrida, E. Rizzarelli and G. Vecchio, *J. Inorg. Biochem.* **50**, 273 (1993).
16. Q.-H. Luo, Q. Lu, A.-B. Dai and L.-G. Huang, *J. Inorg. Biochem.* **51**, 655 (1993).
17. D. Darr, K. A. Zarrilla and I. Fridovich, *Arch. Biochem. Biophys.* **258**, 251 (1987).
18. Y. Nishida, I. Watanabe and K. Unoura, *Chem. Lett.* **1991**, 1517
19. T. Nagano, T. Hirano and M. Hirobe, *J. Biol. Chem.* **264**, 9243 (1989).
20. F. S. Archibald and I. Fridovich, *Arch. Biochem. Biophys.* **214**, 252 (1982).
21. C. J. Feng, Q. H. Luo, Z. L. Wang, M. C. Shen, H. W. Wang and M. H. Zhao, *J. Inorg. Biochem.* **75**, 1 (1999).

22. J. V. Bannister, W. H. Bannister, G: Ratilio, *Crit. Rev. Biochem. Mol.* **22**, 111 (1987).
23. J. M. McCord, *Free Radical Biol. Med.* **4**, 9 (1988).
24. N. Kitajima, *Adv. Inorg. Chem.* **39**, 1 (1992).
25. Q. Lu, C. -Y. Shen and Q. -H. Luo, *Polyhedron* **12**, 2005 (1993).
26. S. Kubota and J. -T. Yang, *Proc. Natl. Acad. Sci. USA* **81**, 3283 (1984).
27. R. P. P. Bonomo, E. Conte, G. Impellizzeri, G. Pappalardo, R. Purrello and E. Rizzarelli, *J. Chem. Soc., Dalton Trans.* **1996**, 3093
28. J. Muller, K. Felix, C. Maichle, E. Lengfelder, J. Strahle and U. Weser, *Inorg. Chim. Acta.* **233**, 11 (1995).
29. J. Casanova, G. Alzuet, J. Borras and O. Carugo, *J. Chem. Soc., Dalton Trans.* **1996**, 2239
30. Q. Lu, Q. -H. Luo, A. -B. Dai, Z. -Y. Zhou, G. -Z. Hu, *J. Chem. Soc. Commun.* **1990**, 1430
31. J. L. Pierre, P. Chautemps, S. Refaif, S. M. Beguin, A. E. Marzouki, G. Serratrice, E. Saint-Aman and P. Rey, *J. Am. Chem. Soc.* **117**, 1965 (1995).
32. R. J. Burdon, V. Gill and C. Rice-Evans, *Free Rad. Res. Comms.* **11**, 65 (1990).
33. A. M. Michelson, G. Jadot and K. Puget, *Free Rad. Res. Comms.* **4**, 209 (1988).
34. J. Emerit, S. Pelletier, D. Tosoni-Verlingue and M. Mollet, *Free Radical Biol. Med.* 7, 145 (1989).
35. S. N. Giri and H. P. Misra, Mol. Biol. **62**, 285 (1984).
36. J. S. Beckman, R. L. Minor, C. W. White, J. Repine, M. Rosen and B. Freeman, *J. Biol. Chem.* **263**, 6884 (1988).
37. J. Schalkwijk, W. B. Van den Berg, L.B.A. Van de Putte, L. A. B. Joosten and L. Van den Bersselaar, *J. Clin. Invest.* **76**, 198 (1985).
38. G. D. Mao and M. J. Poznanski, *Artif. Cell Artif. Organs* **17**, 229 (1989).
39. R. A. Greenwald, *Drugs Today* **26**, 299 (1990).
40. Y. Ilan, J. Rabani, I. Fridovich and R. F. Pasternack, *Inorg. Nucl. Chem. Lett.* **17**, 93 (1981).
41. J. Weinstein and B. H. J. Bielski, *J. Am. Chem. Soc.* **102**, 4916 (1980).
42. L. R. Pickart, Brit. U. K. Pat., Appl. GB. **2**, 213.060, (1987).
43. U. Weser, H. J. Hartmann and R. Miesel, German Patent, Appl. P3920826.5 (1991).
44. G. Czapski and S. Goldstein, *Free Rad. Res. Comms.* **4**, 225 (1988).
45. U. Weser, R. Miesel and M. Linss, in *Antioxidants in Therapy and Preventive Medicine*, I. Emerit (Ed.), Plenum, New York, 1990; p.51
46. Y. Nishida, K. Unoura, I. Watanabe, T. Yokomizo and Y. Kato, *Inorg. Chim. Acta* **181**, 141 (1991).
47. L.L. Costanzo, G. De Guidi, G. Condorelli, A. Cambria and M. Fama, *J. Photochem. Photobiol. B: Biol.* **3**, 223 (1989).
48. A. R. Fersht, *Enzymes, Structure and Mechanism*, Freeman, New York, 2$^{nd}$. ed., 1985.
49. M. Inoue (Ed.), *Protein Engineering: Applications in Science, Industry and Medicine*, Academic Press, New York, 1986.
50. W. F. Beyer, I. Fridovich, G. T. Mullenbach and R. A. Hallewell, *J. Biol. Chem.* **23**, 11182 (1987).
51. L. Banci, I. Bertini, C. Luchinat and R. A. Hallewell, *J. Am. Chem. Soc.* **110**, 3629 (1988).
52. L. Banci, I. Bertini, C. Luchinat and W. Piccioli, *Coord. Chem. Rev.* **100**, 67 (1990).
53. J. A. Tainer, E. D. Getzoff, K. M. Reem, J. S. Richardson and D. C. Richardson, *J. Mol. Biol.* **160**, 181 (1982).

54. A. Cudd and I. Fridovich, *J. Biol. Chem.* **257**, 11443 (1982).
55. L. Banci, D. E. Cabelli, E. D. Getzoff, R. A. Hallewell and M. S. Viezzoli, *J. Inorg. Biochem.* **50**, 89 (1993).
56. A. Petkau, *Cancer Treatment Rev.* **13**, 17 (1986).
57. A. M. Ramadan and M. M. El-Naggar, *J. Inorg.. Biochem.*, **63**, 143 (1996).
58. J. S. Lippard, A. R. Burger, K. Ugurbil, M. W. Pantoliano and J. S. Valentine, *Biochemis.* **16**, 1136 (1977).
59. M. W. Pantoliano, J. S. Valentine, A. R. Burger and J. S. Lippard, *J. Inorg. Biochem.* **17**, 325 (1982)
60. J. A. Tainer, E. D. Getzoff, J. S. Richardson and D. C. Richardson, *Nature* **306**, 284 (1983).
61. M. Linss and V. Weser, *Inorg. Chim. Acta* **138**, 163 (1987).
62. J. Casanova, G. Alzuet, J. Borras, J. Timonneda, S. Garcia-Granda and I. Candano Gonzalez, *J. Inorg. Biochem.* **56**, 65 (1994).
63. A. E. Martin and S. J. Lippard, *J. Am. Chem. Soc.* **106**, 2579 (1984).
64. K. G. Strothkamp and S. J. Lippard, *Acc. Chem. Res.* **15**, 318 (1982).
65. M. G. B. Drew, M. Mac-Cann and S. M. Nelson, *J. Chem. Soc. Dalton Trans* **1981**, 1868.
66. R. Bolli, *Cardiovasc. Drugs Ther.* **5**, 249 (1991).
67. A. G. Chapman, P. E. Keane, B. S. Meldrum, J. Simiand and J. C. Vernieres, *Prog. Neurobiol.* **19**, 325 (1982).
68. J. R. J. Sorenson, *Prog. Med. Chem.* **26**, 437 (1989).
69. P. C. Christidis, P. J. Rentzeperis, M. S. Sigalas and C. C. Hadjikostas, *Z. Kristallogr.* **176**, 103 (1986).
70. J. Catterick and P. Thornton, *Adv. Inorg. Chem. Radiochem.* **20**, 291 (1977).
71. U. Weser, K. H. Sellinger, E. Lengfelder, W. Werner and J. Strahle, *Biochim. Biophys. Acta* **631**, 232 (1980).
72. U. Deuschle and U. Weser, *Inorg. Chim. Acta* **91**, 237 (1984).
73. W. I. Sundquist and S. J. Lippard, *Coord. Chem. Rev.* **100**, 293 (1990).
74. Y. Yorkovsky and B: L. Silver, *J. Inorg. Biochem.* **65**, 35 (1997).
75. D. E. Ose and I. Fridovich, *Biol. Chem.* **251**, 1217 (1976).
76. F. Yamakura and K. Suzuki, *J. Biochem. (Tokyo)* **88**, 191 (1980).
77. M. E. Martin, B. R. Byers, M. O. Olson, M. L. Salin, J. E. L. Arceneaux and C. Tolbert, *J. Biol. Chem.* **261**, 9361 (1986).
78. T. Matsumoto, K. Terauchi, T. Isobe, K. Matsuoka and F. Yamakura, *Biochem.* **30**, 3210 (1991).
79. W. F. Beyer and I. Fridovich, *Biochem.* **26**, 1251 (1987).
80. A. L. Abuhijleh, *J. Inorg. Biochem.* **68**, 167 (1997).
81. D. P. Riley, *Chem. Rev.* **99**, 2573 (1999).
82. Y. Kira E. F. Sato and M. Inoue, *Arch. Biochem. Biophys.*, **399** , 96 (2002).
83. P. J. Hart, M. M. Balbirnie, N. L. Ogihara, A. M. Nersissian, M. S. Weiss, J. S. Valentine and D. Eisenberg, *Biochem.* **38** , 2167 (1999).
84. P. Cioni, M. E. Stroppolo, A. Desideri and G. B. Strambini, *Arch. Biochem. Biophys*, **391**, 111 (2001).
85. H. Ohtsu, S. Itoh, S. Nagatomo, T. Kitagawa, S. Ogo, Y. Watanabe and S. Fukuzumi, *Chem Commun.*, **2000**, 1051.

